# Blocking ERK-DAPK1 Axis Attenuates Glutamate Excitotoxicity in Epilepsy

**DOI:** 10.3390/ijms23126370

**Published:** 2022-06-07

**Authors:** Chen-Ling Gan, Yulian Zou, Dongmei Chen, Xindong Shui, Li Hu, Ruomeng Li, Tao Zhang, Junhao Wang, Yingxue Mei, Long Wang, Mi Zhang, Yuan Tian, Xi Gu, Tae Ho Lee

**Affiliations:** 1 Fujian Key Laboratory of Translational Research in Cancer and Neurodegenerative Diseases, Institute for Translational Medicine, School of Basic Medical Sciences, Fujian Medical University, Fuzhou 350122, China; ganchenling@163.com (C.-L.G.); dmchen88@fjmu.edu.cn (D.C.); xindongshui18@163.com (X.S.); lhu0701@163.com (L.H.); hupimao@live.com (R.L.); taozh@fjmu.edu.cn (T.Z.); wjh15186289445@163.com (J.W.); mln2963@163.com (Y.M.); wangjameslong@163.com (L.W.); zm15861353835@126.com (M.Z.); tian18615526627@163.com (Y.T.); guxi124@126.com (X.G.); 2 Fujian Provincial Key Laboratory of Natural Medicine Pharmacology, Institute of Materia Medica, School of Pharmacy, Fujian Medical University, Fuzhou 350122, China; 3 Immunotherapy Institute, Fujian Medical University, Fuzhou 350122, China; zouyl1234@163.com

**Keywords:** apoptosis, death-associated protein kinase 1 (DAPK1), extracellular signal-regulated kinase (ERK), glutamate excitotoxicity, kainic acid, seizure

## Abstract

Glutamate excitotoxicity induces neuronal cell death during epileptic seizures. Death-associated protein kinase 1 (DAPK1) expression is highly increased in the brains of epilepsy patients; however, the underlying mechanisms by which DAPK1 influences neuronal injury and its therapeutic effect on glutamate excitotoxicity have not been determined. We assessed multiple electroencephalograms and seizure grades and performed biochemical and cell death analyses with cellular and animal models. We applied small molecules and peptides and knocked out and mutated genes to evaluate the therapeutic efficacy of kainic acid (KA), an analog of glutamate-induced neuronal damage. KA administration increased DAPK1 activity by promoting its phosphorylation by activated extracellular signal-regulated kinase (ERK). DAPK1 activation increased seizure severity and neuronal cell death in mice. Selective ERK antagonist treatment, DAPK1 gene ablation, and uncoupling of DAPK1 and ERK peptides led to potent anti-seizure and anti-apoptotic effects in vitro and in vivo. Moreover, a DAPK1 phosphorylation-deficient mutant alleviated glutamate-induced neuronal apoptosis. These results provide novel insight into the pathogenesis of epilepsy and indicate that targeting DAPK1 may be a potential therapeutic strategy for treating epilepsy.

## 1. Introduction

Epilepsy is a chronic neurological disorder. Approximately 70 million people worldwide suffer from seizures with epilepsy, stroke, traumatic brain injury, or Alzheimer’s disease (AD), leading to a great economic burden on families, societies, and governments [1]. Temporal lobe epilepsy (TLE) is a common type of refractory epilepsy in adults and is traditionally considered to be focal epilepsy [2]. TLE patients present with cognitive defects, decreased attention, and functional connectivity decline as well as seizures [3,4,5]. TLE significantly interferes with the quality of daily life of patients because these patients often have cognitive, psychiatric, and behavioral problems that affect work and relationships [6]. From the perspective of clinical practice, TLE is currently particularly challenging to treat owing to frequent resistance to multiple traditional antiepileptic drugs [7]. Although surgical excision of seizure foci is a relatively successful approach for controlling epilepsy in many cases, TLE patients who undergo surgery often show poor seizure control [8,9,10,11]. Moreover, traditional first-line medication is often ineffective in TLE patients [12,13].

Current medications used for TLE only offer symptomatic relief and typically induce adverse drug reactions. Carbamazepine, a commonly used drug for the treatment of TLE, is reported to reduce atrioventricular conduction, thereby increasing the risk of arrhythmias, especially in patients with interictal cardiac autonomic dysfunction [14,15,16,17,18]. Moreover, carbamazepine treatment has shown neurotoxic effects, such as dysphasia [19,20]. Valproic acid is mainly used for the control of generalized tonic-clonic seizures (GTCSs), which have a tonic phase followed by clonic muscle contractions and are the most severe form of common epileptic seizure, and status epilepticus; however, valproic acid often causes teratogenic effects in women and severe hepatotoxicity [21,22,23,24].

Death-associated protein kinase 1 (DAPK1) is a calcium/calmodulin-dependent serine/threonine kinase that plays a crucial role in neuronal function and cell death [25,26,27,28,29]. DAPK1 might play essential roles in neurogenesis, synaptic transmission, and plasticity because it is relatively overexpressed in the cerebral cortex and hippocampus and in cerebellar Purkinje cells in the developmental stage, and its expression declines and is confined to cortical and hippocampal areas in adult brains [30]. Moreover, dysregulation of DAPK1 has been observed in multiple brain degenerative diseases, such as AD [29,31]. Depletion of DAPK1 expression and deletion of the kinase domain of DAPK1 in mice protects against neuronal and spinal damage caused by stroke, respectively [32,33]. DAPK1 knockdown exerts rapid antidepressant-like effects, and DAPK1 ablation in dopaminergic neurons reverses abnormalities in mouse Parkinson’s disease (PD) models after long-term 1-methyl-4-phenyl-1,2,3,6-tetrahydropyridine (MPTP) exposure [34,35]. Moreover, a small molecule that inhibits DAPK1 activity attenuates AD-related neuropathology in cell and animal models [36,37,38,39]. In summary, DAPK1 may serve as a critical component, and its inhibition may have therapeutic effects on a variety of neuropsychiatric diseases.

Our group and other groups have shown that DAPK1 may play a critical role in epilepsy pathophysiology. Levels of DAPK1 are significantly increased in brain samples from patients with epilepsy compared with those in control subject samples [40,41]. In animal models, DAPK1 has been shown to interact with GluN2B and regulate absence epilepsy [42]. Furthermore, the interaction between DAPK1 and tumor necrosis factor receptor 1 or p53 promotes brain cell death in glutamate analog kainic acid (KA)-treated animals, which are commonly used to model human TLE [43,44]. However, the upstream pathways regulating DAPK1 activity in KA-induced seizures in animal models are not known. Therapeutic applications targeting DAPK1 in TLE-mimic models have not been investigated.

In the present work, we showed, for the first time, that DAPK1 is activated by extracellular signal-regulated kinase (ERK) by phosphorylating DAPK1 at Ser735 after KA or glutamate administration. Moreover, the ERK-DAPK1 signaling axis plays an important role in regulating neuronal apoptosis triggered by excitotoxicity. Therefore, DAPK1 gene depletion or treatment with a specific ERK inhibitor or a synthetic blocking peptide to uncouple the ERK and DAPK1 interaction can dramatically reduce seizure severity and neuronal cell death induced by excitotoxicity in vitro and in vivo. Thus, our study provides a novel molecular mechanism and therapeutic option for epileptic seizures.

## 2. Results

### 2.1. DAPK1 Is Activated by ERK-Induced Phosphorylation after KA Insult

**Figure 1:**
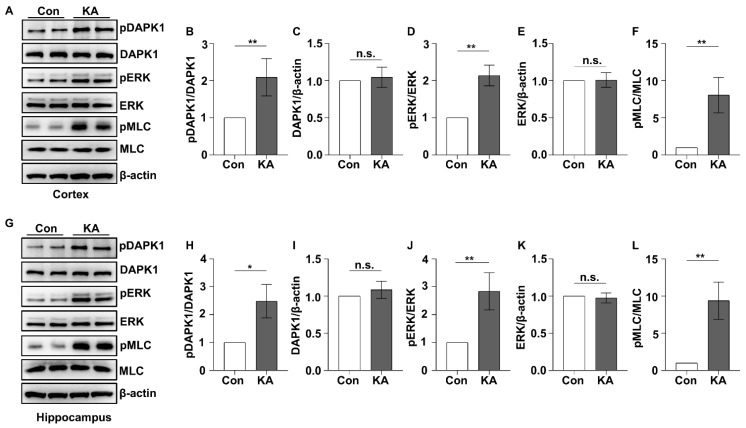
ERK-DAPK1 axis is activated after KA insult. Mice (C57BL/6, male/female, 8–12 weeks, *n* = 7/group) were administered KA (30 mg/kg, i.p.). Then, cortical and hippocampal tissues were dissected 30 min after KA insult. (**A**–**L**) Lysates were resolved on SDS-PAGE gels and then subjected to immunoblotting assays using anti-pSer735-DAPK1, anti-DAPK1, anti-pThr202/Tyr204 ERK, anti-ERK, anti-pSer19-MLC, anti-MLC, or anti-β-actin antibodies (* *p* < 0.05, ** *p* < 0.01 vs. the control; Student’s *t*-test). n.s., not significant (*p* > 0.05). All the values were combined and are expressed as the mean ± SD. Experiments were performed in triplicate with at least two mice per group per experiment.

TLE induced by KA is different from absence epilepsy triggered by pentylenetetrazol (PTZ), with different EEGs, epilepsy phenotypes, and neuronal damage patterns in specific brain regions. Based on the EEG characteristics and behavioral observation, approximately 40–70 min pass before the first GTCS is induced by a single convulsion-inducing dose of KA, but a convulsion-inducing dose of PTZ induces the first GTCS within several minutes [42]. Since DAPK1 expression is upregulated in epilepsy patients, we aimed to examine whether DAPK1 expression and activity are increased by KA before GTCSs. A convulsion-inducing dose of KA (30 mg/kg) was administered to 2- to 3-month-old WT mice, and the brains were harvested 30 min after KA treatment and used in immunoblotting assays. DAPK1 expression was not increased after a single convulsion-inducing dose of KA in either the cortical or hippocampal brain regions, suggesting that KA administration might not regulate DAPK1 protein expression levels (Figure 1A,C,G,I). Next, we examined DAPK1 activity because DAPK1 catalytic activity is regulated by posttranslational modifications [25,28,29]. We were particularly interested in evaluating the phosphorylation of DAPK1 since ERK positively regulates DAPK1 activity by phosphorylating the Ser735 residue of DAPK1 [45] and is activated in multiple epileptic seizures [46,47,48,49,50]. KA administration increased ERK activity more than twofold compared with saline treatment, as normalized by the amount of total ERK (Figure 1A,D,E,G,J,K). The phosphorylation of DAPK1 at Ser735 was induced followed by ERK phosphorylation in the cortex and hippocampus after KA treatment (Figure 1A,B,G,H). Moreover, the activation of DAPK1 was confirmed by the phosphorylation of myosin light chain (MLC), a well-known DAPK1 substrate (Figure 1A,F,G,L) [32,45]. In summary, the ERK-DAPK1 axis in the mouse brain is activated by acute KA treatment.

### 2.2. ERK Inhibitor Reduces DAPK1 Phosphorylation, Seizure Severity and Neuronal Cell Death

**Figure 2:**
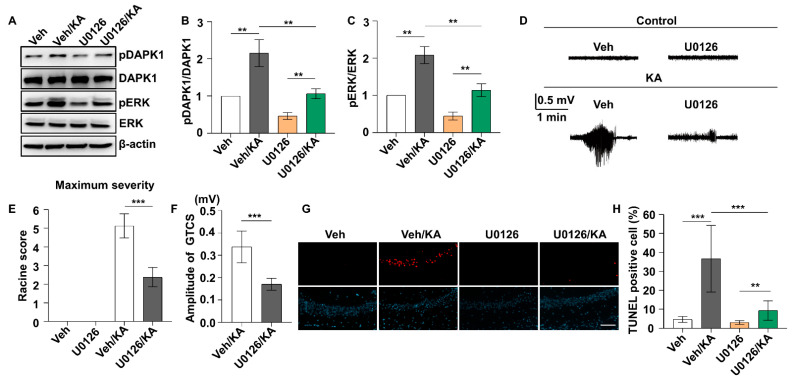
U0126 reduces DAPK1 phosphorylation, seizure severity, and neuronal cell death. Electrodes were implanted into mice (C57BL/6, male/female, 8–12 weeks, *n* = 10/group), which were allowed to recover for 7 days. The mice were treated with the specific ERK inhibitor, U0126 (25 mg/kg, i.p.), or the corresponding vehicle for 180 min before KA insult. Hippocampal tissues were dissected from a number of animals (*n* = 4) 30 min after KA insult for immunoblotting analysis, and EEG traces and behavioral analysis were performed continuously in the remaining mice for 120 min. (**A**–**C**) U0126 intervention suppresses ERK and DAPK1 activity after KA treatment. The brain lysates were resolved on SDS-PAGE gels and then subjected to immunoblotting assays using anti-pSer735-DAPK1, anti-DAPK1, anti-pThr202/Tyr204 ERK, anti-ERK, or anti-β-actin antibodies (** *p* < 0.01, one-way ANOVA with Tukey’s multiple comparisons test). (**D**) Representative EEG recordings from 0.9% saline- and KA-treated mice are displayed as the vehicle- and U0126-treated groups, respectively. (**E**,**F**) U0126-treated mice displayed reduced seizure scales (**E**) and GTCS amplitudes (**F**) after KA administration (*** *p* < 0.001, Student’s *t*-test). (**G**) Representative image showing TUNEL and Hoechst 33,342 staining in the hippocampus is displayed for the vehicle- and U0126-treated groups 72 h after KA treatment. (**H**) Statistical analysis of the TUNEL assay results (** *p* < 0.01, *** *p* < 0.001, one-way ANOVA with Tukey’s multiple comparisons test). Scale bar 100 μm. All the values were combined and are expressed as the mean ± SD. Experiments were performed in triplicate with at least three mice per group per experiment.

Considering that the ERK-DAPK1 axis is activated after KA treatment, we hypothesized that DAPK1 is inhibited by an ERK inhibitor, causing anti-epileptic effects and attenuating neuronal apoptosis induced by KA. To test this hypothesis, the ERK-specific inhibitor U0126 was chosen since it effectively inhibits ERK activity [51,52]. As shown in Figures S1 and S2, U0126 pretreatment for 90 or 180 min significantly reduced the phosphorylation levels of ERK, DAPK1, and MLC, suggesting that inhibition of ERK activity suppressed DAPK1 catalytic activity. WT mice were administered U0126 or vehicle at a dose of 25 mg/kg for 180 min followed by KA treatment. Compared with the vehicle-treated group, the levels of phosphorylated ERK and DAPK1 were effectively reduced after KA insult in the U0126 pretreatment group (Figure 2A–C). Mice treated with KA displayed a series of seizure-like activities, beginning with behavioral arrest (immobility or freezing), followed by repeated head nodding, which progressed to GTCS. Multiple GTCSs were recorded by behavior and EEG traces, and these outcomes were obviously different than the effects induced by administration of a convulsion-inducing dose of PTZ. We found that the ERK inhibitor significantly reduced seizure severity and the amplitude of electrographic seizures (Figure 2D–F). To examine the effect of the ERK inhibitor on KA-induced neuronal injury, a TUNEL assay was performed to measure the neuronal cell apoptosis rate 72 h after KA exposure. While KA insult induced neuronal cell death, the ERK inhibitor dramatically attenuated neuronal apoptosis in the hippocampal region (Figure 2G,H). Collectively, these results indicate that pretreatment with the ERK inhibitor U0126 reduces mouse susceptibility to epilepsy and protects against the neuronal damage induced by KA by inhibiting DAPK1 activity.

### 2.3. Uncoupling DAPK1 from ERK Attenuates KA-Induced Seizures and Neuronal Apoptosis

**Figure 3:**
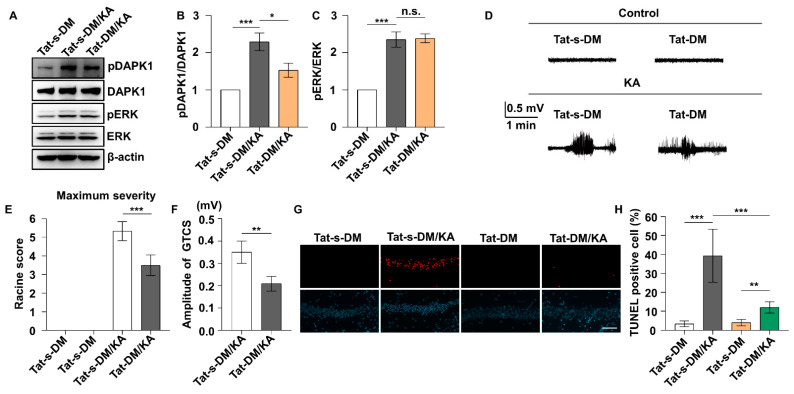
Tat-DM rescues seizure severity and reduces neuronal apoptosis induced by KA. Electrodes were implanted into mice (C57BL/6, male/female, 8–12 weeks, *n* = 10/group), which were allowed to recover for 7 days. The mice were treated with Tat-DM (10 mg/kg, i.p.) or Tat-s-DM for 3 h before KA administration. Hippocampal tissues were dissected from a number of the animals (*n* = 4 per group) 30 min after KA insult for use in immunoblotting assays, and EEG traces and behavioral analysis were continuously performed in the remaining mice for 120 min. (**A**–**C**) Tat-DM intervention suppresses DAPK1 activity after KA treatment. The brain lysates were resolved on SDS-PAGE gels and then subjected to immunoblotting using anti-pSer735-DAPK1, anti-DAPK1, anti-pThr202/Tyr204 ERK, anti-ERK, or anti-β-actin antibodies (* *p* < 0.05, *** *p* < 0.001, one-way ANOVA with Tukey’s multiple comparisons test). (**D**) Representative EEG recordings from 0.9% saline- and KA-treated mice are shown for the Tat-s-DM and Tat-DM groups. (**E**,**F**) Tat-DM-treated mice exhibited reduced seizure scales (**E**) and reduced GTCS amplitudes (F) post KA administration (** *p* < 0.01, *** *p* < 0.001, Student’s *t*-test). (**G**) Representative images showing TUNEL and Hoechst 33,342 staining in the hippocampus in the Tat-s-DM- or Tat-DM-treated groups 72 h after KA treatment. (**H**) Statistical analysis of the TUNEL assay results (** *p* < 0.01, *** *p* < 0.001, one-way ANOVA with Tukey’s multiple comparisons test). Scale bar, 100 μm. n.s., not significant (*p* > 0.05). All the values were combined and are expressed as the mean ± SD. Experiments were performed in triplicate with at least three mice per group per experiment.

**Figure 4:**
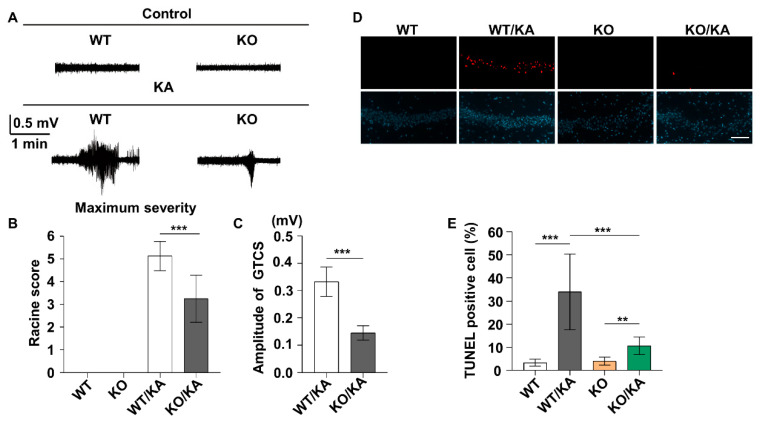
DAPK1 KO attenuates seizure severity and neuronal apoptosis induced by KA. The electrodes were implanted into mice (C57BL/6, male/female, 8–12 weeks, *n* = 8/group), which were allowed to recover for 7 days. WT and DAPK1 KO mice were treated with the same dose of KA. (**A**) Representative EEG recordings from 0.9% saline- and KA-treated mice are shown for WT and DAPK1 KO mice. (**B**,**C**) DAPK1 ablation in mice led to reduced seizure scales (**B**) and GTCS amplitudes (**C**) after KA insult compared with their age-matched WT littermates (** *p* < 0.01, *** *p* < 0.001, Student’s *t*-test). (**D**) Representative images showing TUNEL and Hoechst 33,342 staining in the hippocampus in the WT or KO group 72 h after KA treatment. (**E**) Statistical analysis of the TUNEL assay results (** *p* < 0.01, *** *p* < 0.001, one-way ANOVA with Tukey’s multiple comparisons test). Scale bar, 100 μm. All the values were combined and are expressed as the mean ± SD. Experiments were performed in triplicate with at least two mice per group per experiment.

Because the ERK inhibitor attenuated KA-induced seizure and neuronal cell death and inhibited DAPK1 activity (Figure 2) and because ERK interacts with DAPK1 through a specific ERK-binding site in the death domain of DAPK1 [45], we investigated whether uncoupling DAPK1 from ERK affects seizure phenotypes and the neuronal cell death rate by treating mice with a blocking peptide. A short membrane-permeable peptide was synthesized by fusing a DAPK1-binding peptide, LGRRDAADFLLKAS, to a Tat peptide derived from the HIV TAT protein (Tat-DM) and a scramble control (Tat-s-DM), and the construct was confirmed by HPLC and mass spectrometry (Figure S3). The interaction between ERK and DAPK1 was effectively disrupted by Tat-DM but not control Tat-s-DM peptides (Figure S4A,B). Moreover, the level of phosphorylated DAPK1 at Ser735 was decreased with Tat-DM peptides (Figure S4C–H). In the KA mouse model, Tat-DM treatment reduced DAPK1 activity without affecting ERK activity, confirming that DAPK1 is a substrate of ERK (Figure 3A–C). Moreover, Tat-DM significantly reduced seizure severity and lowered the amplitude of electrographic seizures, but Tat-s-DM did not have the same effects in the KA-induced seizure model (Figure 3D–F). Furthermore, blocking the interaction of ERK and DAPK1 attenuated neuronal apoptosis induced by KA (Figure 3G,H). These results indicate that blocking the binding of ERK and DAPK1 by Tat-DM reduced DAPK1 activity, thereby decreasing susceptibility to epilepsy and attenuating neuronal damage induced by KA. We further studied the effects of DAPK1 on seizure behavior and brain damage in KA-induced epilepsy mouse models on a DAPK1 KO background. DAPK1 deficiency resulted in a lower seizure grade and amplitude of electrographic seizures compared with WT littermates (Figure 4A–C). Because DAPK1 KO decreases susceptibility to epilepsy, we next aimed to examine whether DAPK1 KO protects against neuronal damage induced by KA. DAPK1 KO displayed a potent protective effect on neuronal cell death after epilepsy (Figure 4D,E). Taken together, these results suggest that uncoupling DAPK1 from ERK or knocking out DAPK1 attenuates seizure severity and reduces neuronal apoptosis induced by KA.

### 2.4. The ERK-DAPK1 Axis Is Activated after Glutamate Exposure

**Figure 5:**
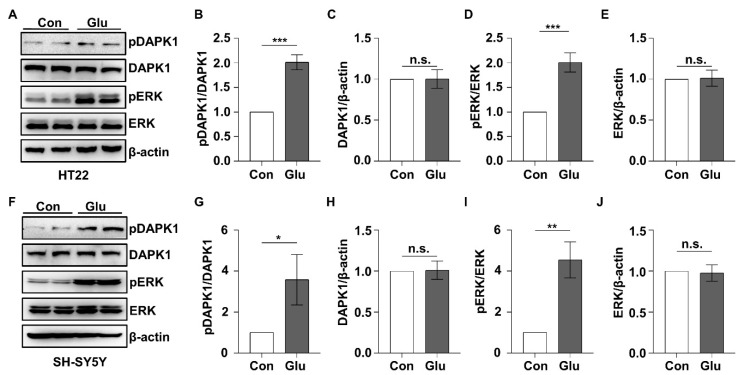
DAPK1 is activated after glutamate exposure. HT22 and SH-SY5Y cells were treated with glutamate (10 mM), and cells were harvested 30 min after glutamate administration. (**A**–**J**) HT22 or SH-SY5Y cell lysates were resolved on SDS-PAGE gels and then subjected to immunoblotting assays using anti-pSer735-DAPK1, anti-DAPK1, anti-pThr202/Tyr204 ERK, anti-ERK, or anti-β-actin antibodies (* *p* < 0.05, ** *p* < 0.01, *** *p* < 0.001 vs. the control; two-tailed Student’s *t*-test). n.s., not significant (*p* > 0.05). All data represent the mean ± SD of three independent experiments.

**Figure 6:**
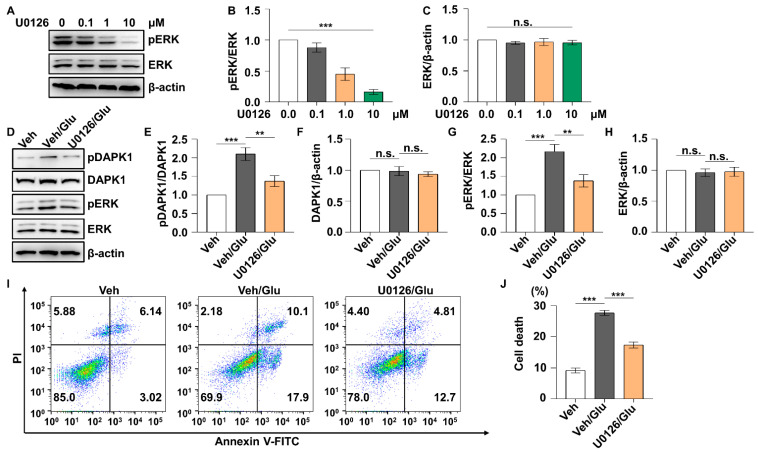
ERK inhibitor reduces DAPK1 activity and apoptosis induced by glutamate. HT22 cells were incubated with 0, 0.1, 1.0, and 10 μM U0126 for 60 min. (**A**–**C**) HT22 cell lysates were resolved on SDS-PAGE gels and then subjected to immunoblotting assays with anti-pThr202/Tyr204 ERK, anti-ERK, or anti-β-actin antibodies (*** *p* < 0.001; one-way ANOVA with Tukey’s multiple comparisons test). HT22 cells were pretreated with 10 μM U0126 for 60 min followed by washing with PBS. Cells were harvested 1 h after 10 mM glutamate incubation and used in immunoblotting assays using anti-pSer735-DAPK1, anti-DAPK1, anti-pThr202/Tyr204 ERK, anti-ERK, or anti-β-actin antibodies (**D**–**H**), and apoptosis was detected 24 h after glutamate treatment by flow cytometry (**I**,**J**) (** *p* < 0.01, *** *p* < 0.001; one-way ANOVA followed by Tukey’s multiple comparisons test). n.s., not significant (*p* > 0.05). All data represent the mean ± SD of three independent experiments.

Accumulating evidence suggests that excitotoxicity induced by glutamate may account for neuronal damage in epilepsy [53,54]. We aimed to investigate the role of the ERK-DAPK1 signaling pathway in glutamate-induced neuronal apoptosis with cell models. We chose to use the HT22 mouse hippocampal neuronal cell line, which is highly sensitive to glutamate, and the SH-SY5Y human neuroblastoma cell line. The phosphorylation level of ERK was significantly increased after 10 mM glutamate treatment of both HT22 (Figure 5A,D) and SH-SY5Y cell lines (Figure 5I). The phosphorylation of DAPK1 at Ser735 was also induced after glutamate exposure in both HT22 and SH-SY5Y cells (Figure 5A,B,F,G). The total levels of ERK and DAPK1 remained unchanged (Figure 5A,C,E,F,H,J). U0126, an ERK-specific inhibitor, inhibited ERK activity in a dose-dependent manner in HT22 cells (Figure 6A–C). To examine whether the ERK-DAPK1 axis is involved in glutamate-induced neuronal damage, HT22 cells were incubated with U0126 for 1 h, and then, glutamate was added to the cells and incubated for 24 h. The phosphorylation level of DAPK1 was reduced after glutamate insult of U0126-pretreated cells, showing reduced ERK phosphorylation (Figure 6D–H). Moreover, U0126 pretreatment reduced the glutamate-induced neuronal cell death rate (Figure 6I,J). In summary, the ERK-DAPK1 axis is activated after glutamate insult in different neuronal cell lines.

### 2.5. Tat-DM- or DAPK1 Phosphorylation-Deficient Forms Attenuate Neuronal Apoptosis

**Figure 7:**
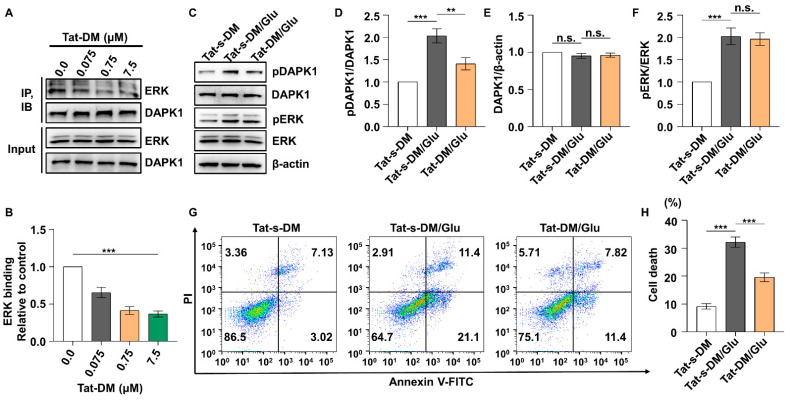
Uncoupling DAPK1 from ERK reverses neuronal apoptosis induced by glutamate. HT22 cells were incubated with 0, 0.075, 0.75, and 7.5 μM Tat-DM for 180 min. (**A**,**B**) HT22 cell lysates were immunoprecipitated with DAPK1 antibody, resolved on SDS-PAGE gels and subjected to immunoblotting assays using anti-DAPK1 or anti-ERK antibodies (*** *p* < 0.001; one-way ANOVA with Tukey’s multiple comparisons test). HT22 cells were pretreated with 7.5 μM Tat-DM for 180 min followed by washing with PBS. Cells were harvested 1 h after 10 mM glutamate incubation and used in immunoblotting assays with anti-pSer735-DAPK1, anti-DAPK1, anti-pThr202/Tyr204 ERK, anti-ERK, or anti-β-actin antibodies (**C**–**F**), and apoptosis was detected 24 h after glutamate treatment by flow cytometry (**G**,**H**) (** *p* < 0.01, *** *p* < 0.001; one-way ANOVA with Tukey’s multiple comparisons test). n.s., not significant (*p* > 0.05). All data represent the mean ± SD of three independent experiments.

**Figure 8:**
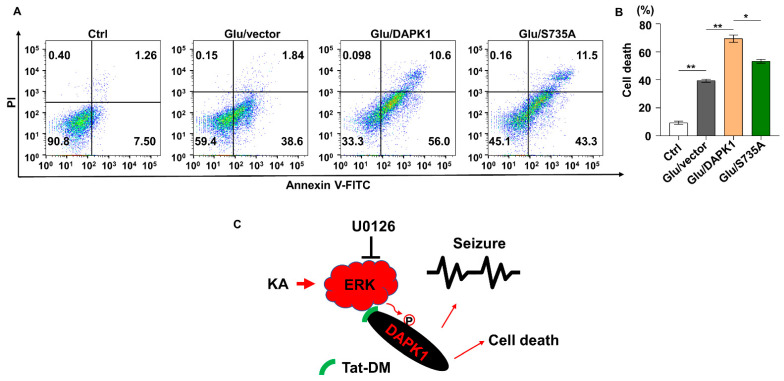
Increased glutamate-induced apoptosis is alleviated by the DAPK1 mutant. (**A**,**B**) Flag-Vector, Flag-DAPK1, or Flag-DAPK1-S735A was transfected into SH-SY5Y cells for 36 h followed by 10 mM glutamate treatment, and then the apoptosis rate was measured 24 h after glutamate incubation by flow cytometry (* *p* < 0.05, ** *p* < 0.01; one-way ANOVA with Tukey’s multiple comparisons test). All data represent the mean ± SD of three independent experiments. (**C**) Schematic representation of the proposed model of the ERK-DAPK1 axis in neuronal injuries induced by glutamate excitotoxicity.

Because ERK-DAPK1 signaling pathways are involved in glutamate-induced neuronal apoptosis, we examined whether blocking the ERK-DAPK1 axis attenuates glutamate toxicity in cells carrying Tat-DM and a phosphorylation-deficient form of DAPK1 (S735A). When a series of Tat-DM concentrations were added to HT22 cells, ERK was uncoupled from DAPK1 in a dose-dependent manner (Figure 7A,B). Moreover, Tat-DM pretreatment reduced phosphorylated DAPK1 levels and cell apoptosis induced by glutamate toxicity, but Tat-s-DM treatment did not have the same effects (Figure 7C–H), indicating that disruption of ERK and DAPK1 binding decreased DAPK1 activity, reducing glutamate-triggered cell death. Next, we introduced a DAPK1 phosphorylation-deficient form in which alanine replaced serine (Figure S5). As shown in Figure 8A,B, DAPK1 overexpression significantly facilitated glutamate-induced cell death, while the S735A mutant markedly reduced cell death, suggesting that phosphorylation of DAPK1 at Ser735 by ERK contributes to DAPK1-mediated apoptosis treated with glutamate.

## 3. Discussion

Although TLE is the most common form of focal epilepsy in adults and people with TLE are at high risk for memory loss, the molecular mechanisms of TLE and potential therapeutic strategies are largely unknown. In the present study, we used KA-injected mice as in vivo models and glutamate-treated neuronal cell lines as in vitro models to study the signaling pathways of ERK and DAPK1 during seizures and in the context of neuronal survival. We discovered that ERK activation by KA or glutamate promoted DAPK1 kinase activity through its phosphorylation at Ser735, progressing to GTCS and increased neuronal death. The application of a specific ERK inhibitor or synthetic blocking peptide to bind ERK and DAPK1 reduced susceptibility to epilepsy and protected neurons from apoptosis in both in vitro and in vivo models. Moreover, DAPK1 KO mice or DAPK1 S735A mutant-expressing neuronal cells showed markedly alleviated seizure severity and reduced neuronal apoptosis. Collectively, the ERK-DAPK1 signaling axis might contribute to neuronal injuries triggered by glutamate excitotoxicity in TLE.

Elevated ERK activity has been shown in several seizure models, including audiogenic seizures in Fragile X syndrome, KA-induced seizures, and electric stimulation-induced seizures [55,56,57]. ERK phosphorylation in neurons is one of the earliest seizure indicators, and it is induced after a spontaneous seizure within very short intervals: 1 or 2 min [58]. Moreover, ERK activation induced by the overexpression of the constitutively active form of MEK1 directly triggered spontaneous epileptic seizures via upregulation of NMDA receptor activity [46]. ERK was subsequently found to contribute to the onset of epilepsy by activating downstream kinases, including RSK and MAPK-interacting kinase 1 [55]. Although the role of ERK activation and the effect of ERK inhibitors on neuronal cell death triggered by glutamate showed conflicting results, increasing evidence has suggested that ERK inhibition attenuates the effect in epilepsy-induced cell death [47,48,59,60,61]. In the current study, an ERK inhibitor not only reduced susceptibility to epilepsy but also protected neurons from excitotoxicity by suppressing DAPK1 activity, indicating that DAPK1 may be a downstream effector of ERK in KA-induced epilepsy models.

ERK binds to DAPK1 through the death domain of DAPK1, which contains binding sequences required for the interaction [45]. Moreover, ERK directly phosphorylates DAPK1 at Ser735 and promotes its apoptotic effect [45]. Our data showed that DAPK1 overexpression resulted in a more than twofold increase in cell death compared with the control in glutamate-treated cells. However, the phosphorylation-deficient form of DAPK1 Ser735, S735A attenuated glutamate-induced cell death in vitro. Furthermore, we observed for the first time that the synthetic blocking peptide Tat-DM markedly uncoupled ERK from DAPK1 and abolished the phosphorylation of DAPK1, suggesting that the ERK docking site is required for the phosphorylation and activation of DAPK1. Tat-DM reduced neuronal apoptosis induced by glutamate excitotoxicity both in vitro and in vivo, indicating that uncoupling ERK from DAPK1 reduced DAPK1 activity and conferred protection against glutamate excitotoxicity. Since DAPK1 activation has also been previously shown to inhibit ERK nuclear localization and suppress ERK-mediated proliferation and antiapoptotic function [45], it would be interesting to determine whether the DAPK1-ERK interplay forms a regulatory circuit in the brain that is induced by glutamate excitotoxicity in epileptic seizures.

DAPK1 has been shown to be involved in various neurodegenerative diseases, such as AD, ischemic stroke, PD and epilepsy [32,33,34,35,36,37,38,39,40,41,42,43,44,62]. DAPK1 expression or activity is upregulated, and DAPK1 KO leads to a protective effect against neurodegenerative diseases. DAPK1 regulates its downstream targets, such as tau, amyloid precursor protein, α-synuclein, and NR2B, as it serves mostly as a direct kinase in the development of neurodegenerative diseases, suggesting that DAPK1 might be a central regulator in these diseases. Although DAPK1 activity is regulated by posttranslational modifications, including phosphorylation, its upstream direct kinase has not been identified. Since we report here, for the first time to our knowledge, that ERK is an upstream regulator of DAPK1 activity in TLE, further studies are warranted to determine whether ERK is required for DAPK1 activation in other neurodegenerative diseases, including different subtypes of epilepsy. Moreover, it is also necessary to identify DAPK1 targets and interacting proteins in KA- and glutamate-induced models to decipher the precise regulatory mechanisms.

In summary, we discovered that KA promotes DAPK1 phosphorylation on Ser735 and induces its activity via ERK activation, thereby increasing excitotoxicity, epileptic seizures, and neuronal cell death in mice. Moreover, we found that glutamate treatment activates the ERK-DAPK1 axis in different neuronal cell lines. Consequently, application of an ERK inhibitor and a synthetic blocking peptide to prevent ERK from binding to DAPK1 reduces DAPK1 phosphorylation and neuronal apoptosis and inhibits the severity of seizures induced by KA or glutamate in in vitro and in vivo models. Furthermore, knocking out the DAPK1 gene exerts a protective effect in the epileptic mouse models (Figure 8C). Our findings thus provide novel insights into the key roles of the ERK and DAPK1 molecular pathways in modulating seizures and suggest new therapeutic approaches for human epilepsy.

## 4. Materials and Methods

### 4.1. Materials

Glutamate, KA, and an ERK inhibitor (U0126) were obtained from MilliporeSigma (St. Louis, MO, USA). Tat-DM (Tat-LGRRDAADFLLKAS) and Tat-s-DM (Tat-RALGADLDALSKFR, Tat-s-DM) were purchased from Allpeptide (Hangzhou, China).

### 4.2. Animal Models

Wild-type (WT) and DAPK1 knockout (KO) mice (8–12 weeks) on a C57BL/6 background were used for all the animal experiments. DAPK1 KO mouse generation was reported previously [63]. Mice were maintained in a reverse 12 h light/dark cycle with food and water. Experiments involving animals were approved by the Animal Ethics Committee of Fujian Medical University.

### 4.3. Cell Culture

The HT22 mouse hippocampal neuronal cell line (SCC129, MilliporeSigma) was cultured in high-glucose Dulbecco’s modified Eagle’s medium (DMEM), and SH-SY5Y human neuroblastoma cells (CRL-2266, American Type Culture Collection, Manassas, VA, USA) were cultured in DMEM Nutrient Mix F12. Both media contained 100 U/mL penicillin, 100 µg/mL streptomycin, and 10% fetal bovine serum. The cultures were maintained at 37 °C in a 5% CO_2_ incubator.

### 4.4. KA Dosing Regimens

Excitotoxicity was induced by intraperitoneal (i.p.) injection of 30 mg/kg KA, and the mice were observed individually for the next 120 min after KA injection. Seizure grade was calculated using a modified Racine scoring system [64]. Stage 0, normal behavior with controlled breathing; stage 1, immobility or freezing, mouth movements, and chewing with fast breathing; stage 2, head bobbing, and wet dog shakes; stage 3, repeated head nodding or forelimb clonus (forepaw shaking); stage 4, forelimb jerking, and repeated anticlockwise exploration (rearing and jumping) with falling; stage 5, generalized clonic, tonic-clonic seizure (postural loss); and stage 6, status epileptics or death.

### 4.5. Electrode Implantation and EEG Recording

Mice were anesthetized and placed on a stereotaxic apparatus to implant epidural electroencephalogram (EEG) electrodes at the following positions (anteroposterior (AP), +2.0 mm, and mediolateral (ML), +1.5 mm) and a reference electrode (AP, −2.0 mm, and ML, +3.0) as described previously [42,65]. Video EEG recordings were taken of freely moving mice to trace seizure behaviors and obtain EEGs. EEGs were acquired through a BL-420F Data Acquisition System at a sampling rate of 5 kHz. EEG signals were continuously recorded for 120 min after KA induction. All EEG signals were processed between 0 and 300 Hz. EEG signals were amplified, filtered, and stored on the computer for off-line analysis. Before KA insult, baseline EEGs were recorded for each animal for 5 min and EEGs were continuously recorded for 120 min after KA induction. A GTCS was identified as a train of spike discharges lasting more than 15 s with an amplitude of at least 2× the initial baseline amplitude, as previously described [66]. All EEG signals were processed between 10 and 300 Hz.

### 4.6. TUNEL Assay

A terminal deoxynucleotidyl transferase-mediated dUTP nick-end labeling (TUNEL) assay was conducted to investigate neuronal cell death caused by KA exposure using an in situ cell death detection kit (Roche, Indianapolis, IN, USA). Briefly, brain sections were rinsed with PBST and immersed in 20 μg/mL proteinase K solution for 20 min at 37 °C. Then, slides were washed at least five times with PBST followed by incubation with TUNEL reaction mixture (enzyme solution:label solution = 1:9) for 90 min at 37 °C. Next, Hoechst 33,342 was added for nuclear staining. Following a final rinse, slides were mounted using Fluoromount-G solution (SouthernBiotech, Birmingham, AL, USA) and photographed with a fluorescence microscope (Zeiss Axio Imager M2, Oberkochen, Germany).

### 4.7. Cell Apoptosis Assay

SH-SY5Y and HT22 cells were harvested at the indicated time points and washed three times with PBS buffer. Then, 1× Annexin V binding buffer was added to resuspend the cells, and fluorescein-labeled Annexin V and nuclear staining solution (propidium iodide) were added. The apoptosis rate was determined from flow cytometry data, and statistical analysis was performed with built-in FlowJo software. Apoptosis rate (%) = (early apoptotic cells + late apoptotic cells)/all cells ×100%.

### 4.8. Immunoprecipitation and Immunoblotting Analysis

Immunoprecipitation and immunoblotting analyses were conducted as previously reported [31,42,67]. Briefly, mouse cortical and hippocampal tissues were lysed using RIPA lysis buffer in the presence of protease and phosphatase inhibitor cocktails. Equal amounts of protein samples from different groups were separated by SDS-PAGE and transferred to 0.45 μm polyvinylidene fluoride membranes. The membranes were blocked with 5% milk-TBST or bovine serum albumin (BSA)-TBST for 1 h and then incubated with different antibodies overnight followed by horseradish peroxidase (HRP)-conjugated secondary antibodies. The protein intensities were detected by ChemiDoc XRS+ and analyzed by densitometry using the Fiji/ImageJ Coloc 2 plug-in. Data were normalized to the corresponding protein bands. For the immunoprecipitation analysis, prepared brain tissue homogenate or cell homogenate was added to primary antibodies and incubated overnight at 4 °C, and then, protein A/G Sepharose beads were added to the mixture (Santa Cruz Biotechnology, Dallas, TX, USA). The beads were washed at least five times with 1× PBS and then denatured with SDS-PAGE sample loading buffer and used in an immunoblot assay. The primary antibodies that were used included anti-pS735 DAPK1 (Thermo Fisher, Waltham, MA, USA; PA5-105872; 1:1000), anti-DAPK1 (MilliporeSigma; D2178; 1:1000), anti-pThr202/Tyr204 ERK (Cell Signaling Technology, Danvers, MA, USA; 9101; 1:5000), anti-ERK (Cell Signaling Technology; 4695; 1:5000), anti-pS19 MLC (Cell Signaling Technology; 3675; 1:1000), anti-MLC (Abcam, Waltham, MA, USA; ab233152; 1:1000), and anti-β-actin (MilliporeSigma; A5441; 1:50,000).

### 4.9. Statistical Analysis

Statistical analysis of all experimental data was conducted using GraphPad Prism 8 software. One-way ANOVA with Tukey’s multiple comparisons test or two-tailed Student’s *t*-test was applied when appropriate. The results are shown as the means ± standard deviation (SD). A *p* value < 0.05 was considered to be significant.

## Data Availability

The datasets generated and/or analyzed in the present study are available from the corresponding author upon reasonable request.

## Supplementary Materials

The following supporting information can be downloaded at: https://www.mdpi.com/article/10.3390/ijms23126370/s1.

## Abbreviations

AD: Alzheimer’s disease; DAPK1, death-associated protein kinase 1; EEG, epidural electroencephalogram; ERK, extracellular signal-regulated kinase; GTCS, generalized tonic-clonic seizure; KA, kainic acid; MLC, myosin light chain; MPTP, 1-methyl-4-phenyl-1,2,3,6-tetrahydropyridine; PTZ, pentylenetetrazol; TLE, temporal lobe epilepsy; TNFR1, tumor necrosis factor receptor 1; TUNEL, terminal deoxynucleotidyl transferase-mediated dUTP nick-end labeling.
