# Supplementary material for: Blocking ERK-DAPK1 Axis Attenuates Glutamate Excitotoxicity in Epilepsy
Source: Int J Mol Sci. 2022 Jun 7;23(12):6370. doi: 10.3390/ijms23126370 (PMC9223430; doi:10.3390/ijms23126370)
Supplement: Supplementary file 1 [file ijms-23-06370-s001.zip › ijms-1701039-supplementary.pdf]

Supplementary Materials

Gan et al.

Blocking ERK-DAPK1 axis attenuates glutamate excitotoxicity in epilepsy

Supplementary Figure Legends

Figure S1. U0126 pretreatment reduces ERK and DAPK1 activity.

Mice (C57BL/6, male/female, 8-12 weeks, n=7 per group) were treated with U0126 (25 mg/kg) and brain tissues were harvested 90 min after administration. **(A-K)** Cortical and hippocampal lysates were subjected to immunoblotting analysis using anti-pSer735-DAPK1, anti-DAPK1, anti-pThr202/Tyr204 ERK, anti-ERK, anti-pSer19-MLC, anti-MLC or anti-β-actin antibodies (**$P < 0.01$, ***$P < 0.001$ vs. the control; two-tailed Student's t-test). n.s., not significant ($P > 0.05$). All the values were combined and are expressed as the mean ± SD. Experiments were performed in triplicate with at least two mice per group per experiment.

Figure S2. U0126 pretreatment decreases ERK and DAPK1 activity.

Mice (C57BL/6, male/female, 8-12 weeks, n=7 per group) were treated with U0126 (25 mg/kg) and brain tissues were harvested 180 min after administration. **(A-K)** Cortical and hippocampal lysates were subjected to immunoblotting analysis using anti-pSer735-DAPK1, anti-DAPK1, anti-pThr202/Tyr204 ERK, anti-ERK, anti-pSer19-MLC, anti-MLC or anti-β-actin antibodies (**$P < 0.01$, ***$P < 0.001$ vs. the control; two-tailed Student's t-test). n.s., not significant ($P > 0.05$). All the values were combined and are expressed as the mean ± SD. Experiments were performed in triplicate with at least three mice per group per experiment.

Figure S3. The information of Tat-DM and Tat-s-DM.

(A-B) High performance liquid chromatography report displays the purity (chromatogram) and relative parameters of the synthesized peptide Tat-DM or Tat-s-DM. **(C-D)** Mass Spectrometry assay shows relative parameters of the synthesized peptide Tat-DM or Tat-s-DM.

Figure S4. Tat-DAPK1DM peptide pretreatment blocks ERK and DAPK1 interaction.

Mice (C57BL/6, male/female, 8-12 weeks, n=7 per group) were treated with Tat-DM peptide (10 mg/kg) and brain tissues were harvested 3 or 6 h after administration. **(A-B)** Hippocampal lysates were immunoprecipitated with non-specific IgM or anti-DAPK1 antibodies and then subjected to immunoblotting analysis using anti-DAPK1 or anti-ERK antibodies (***P < 0.001; two-tailed Student's t-test). **(C-H)** Hippocampal lysates were subjected to immunoblotting analysis with anti-pSer735-DAPK1, anti-DAPK1, anti-pThr202/Tyr204 ERK, anti-ERK, or anti-β-actin antibodies (*P < 0.05, **P < 0.01 vs. the control; two-tailed Student's t-test). n.s., not significant (P > 0.05). All the values were combined and are expressed as the mean ± SD. Experiments were performed in triplicate with at least two mice per group per experiment.

Figure S5. Verification of S735A mutant plasmid.

Flag-DAPK1 or Flag-DAPK1 S735A were transfected to HEK 293T cells for 48 h, and harvested cell lysates were immunoprecipitated with anti-Flag antibody and then subjected to immunoblotting analysis using anti-FLAG, anti-pSer735-DAPK1 or anti-β-actin antibodies.

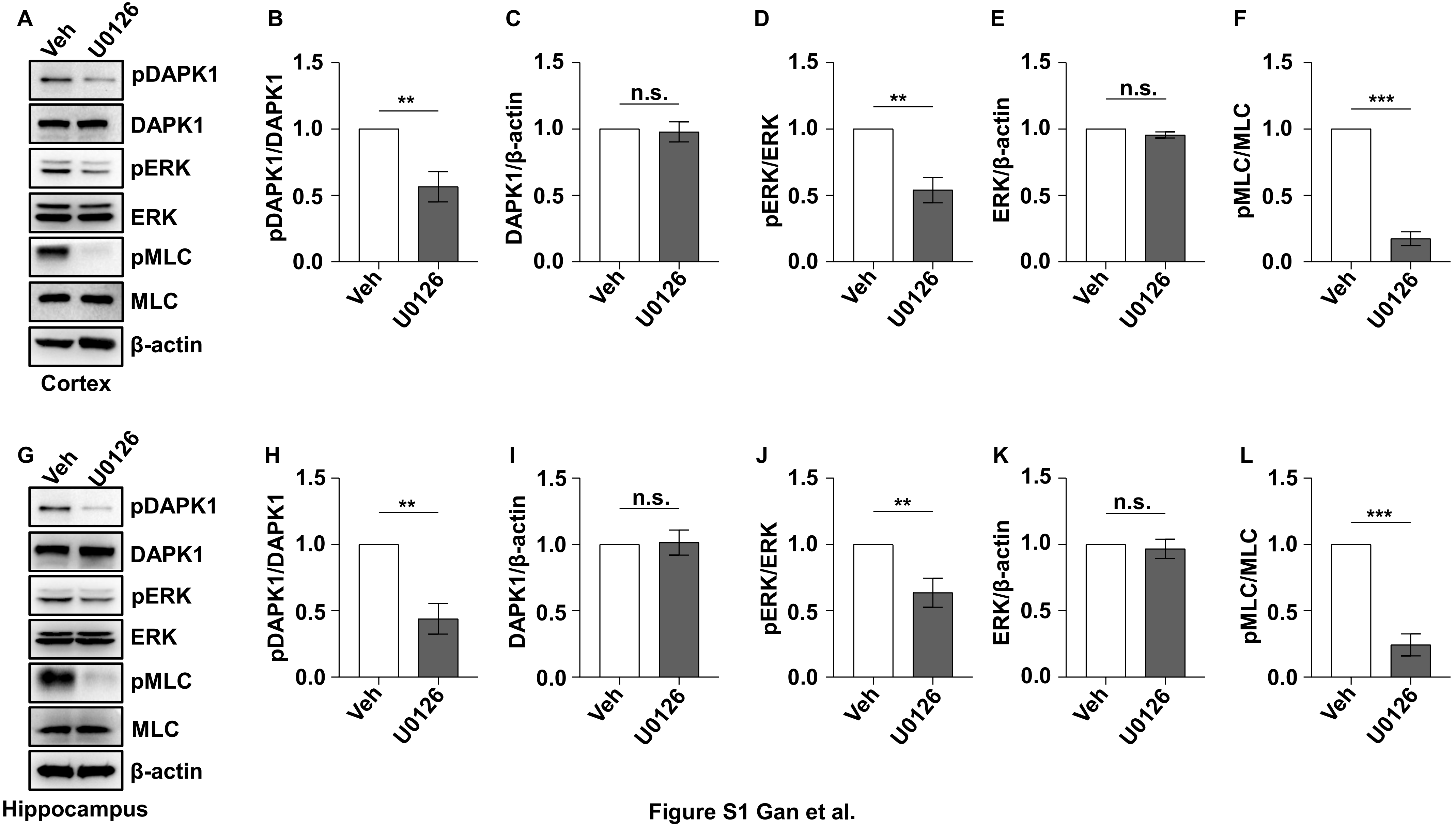

Figure S1 Gan et al.

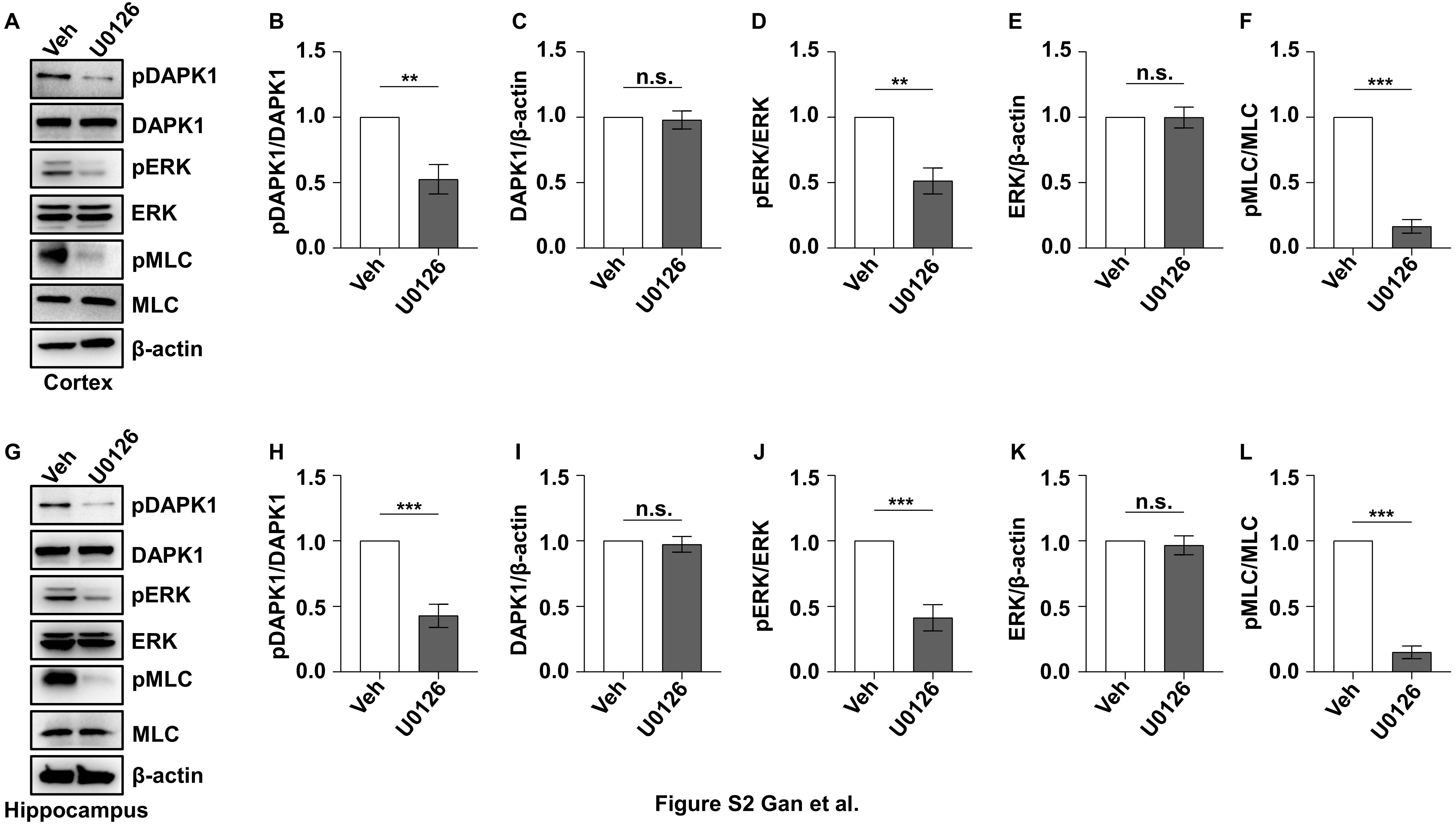

Figure S2 Gan et al.

A

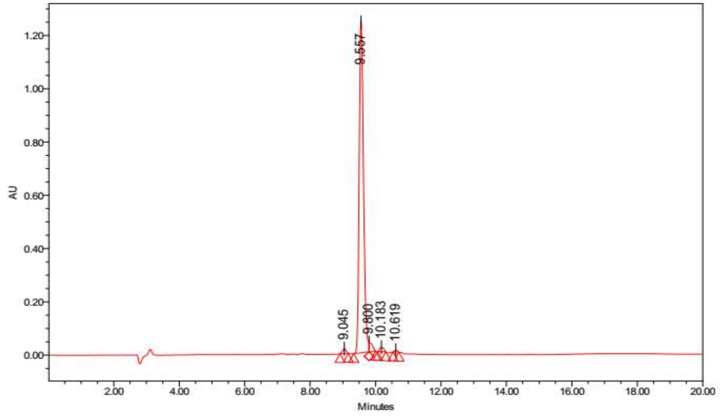

Processed Channel: 2487Channel 1

	Processed Channel	Retention Time (min)	Area	% Area	Height
1	2487Channel 1	9.045	135701	1.13	17961
2	2487Channel 1	9.557	11427221	95.19	1253424
3	2487Channel 1	9.800	248096	2.07	34733
4	2487Channel 1	10.183	146327	1.22	16460
5	2487Channel 1	10.619	47865	0.40	8030

B

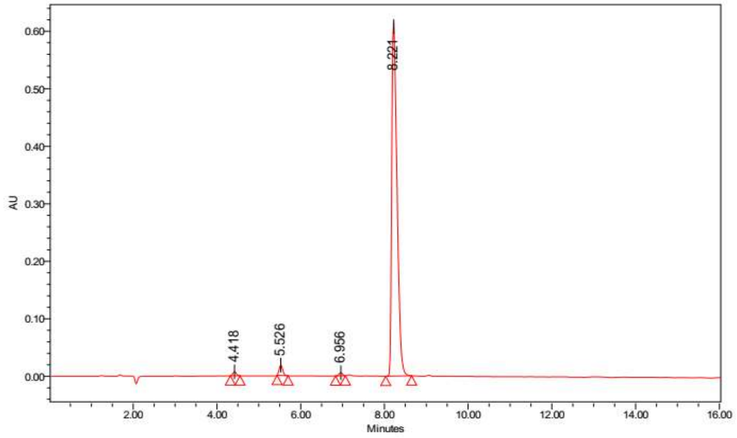

Processed Channel: 2487Channel 1

	Processed Channel	Retention Time (min)	Area	% Area	Height
1	2487Channel 1	4.418	41969	0.76	7098
2	2487Channel 1	5.526	109724	1.97	18031
3	2487Channel 1	6.956	32436	0.58	5674
4	2487Channel 1	8.221	5373246	96.69	616382

C

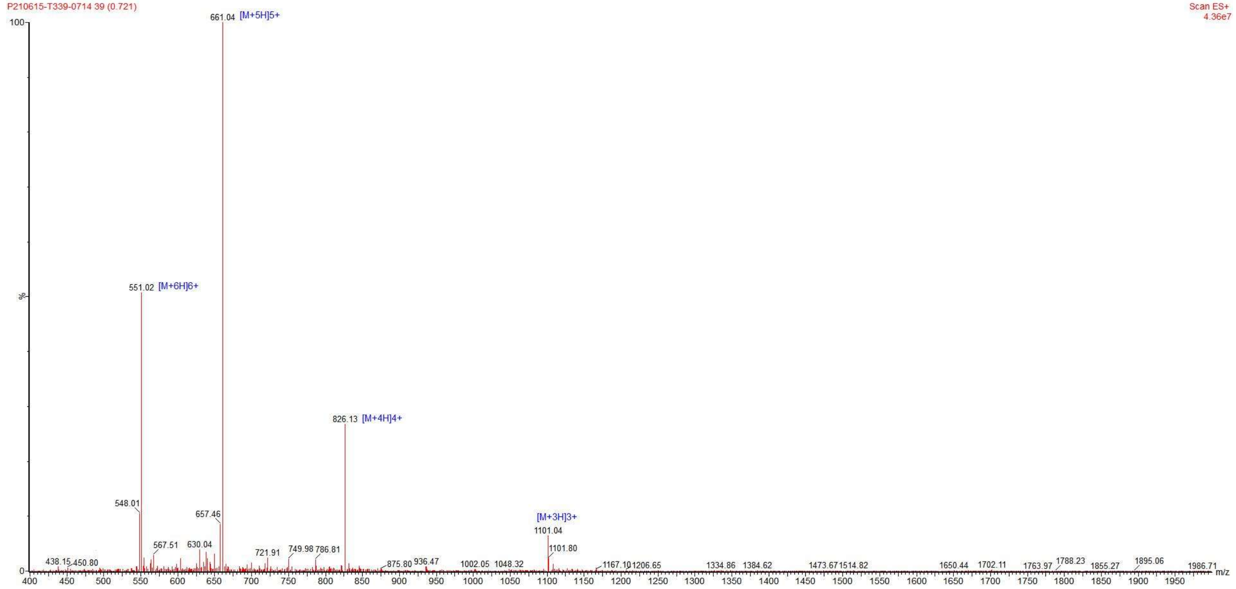

D

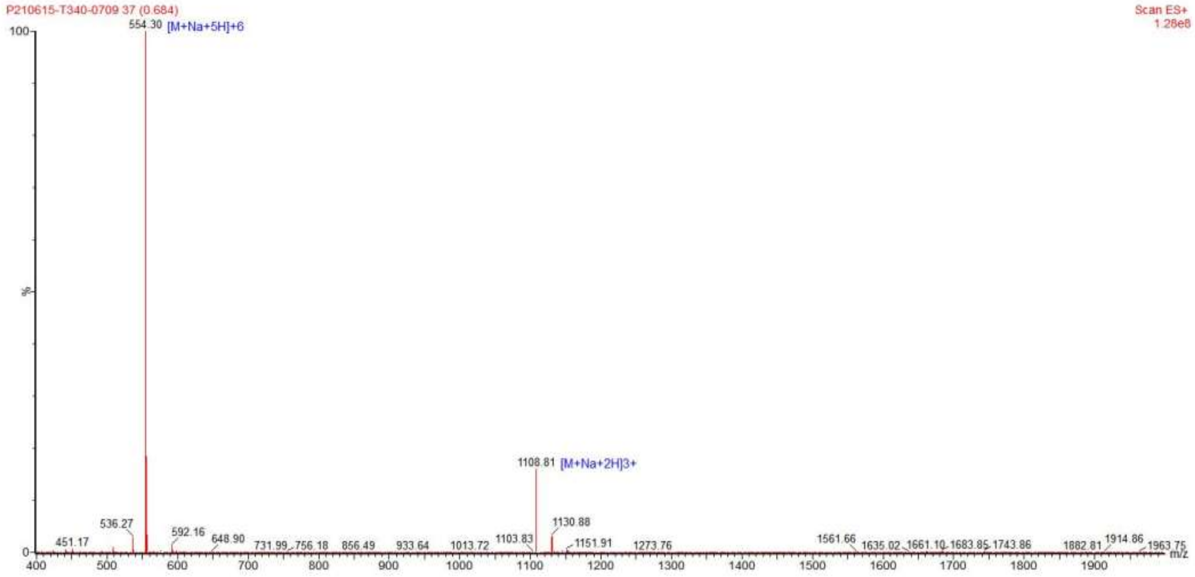

Figure S3 Gan et al.

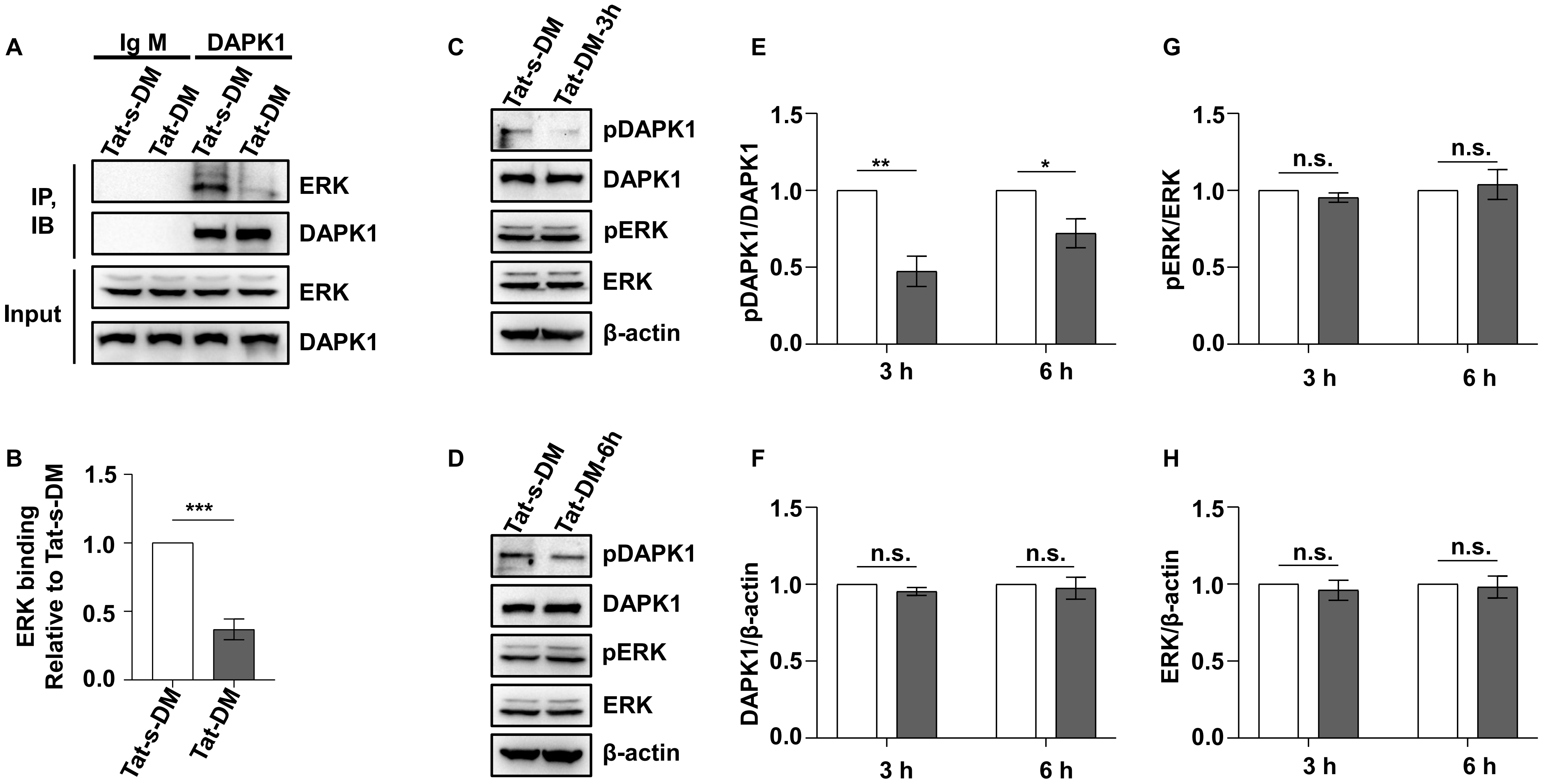

Figure S4 Gan et al.

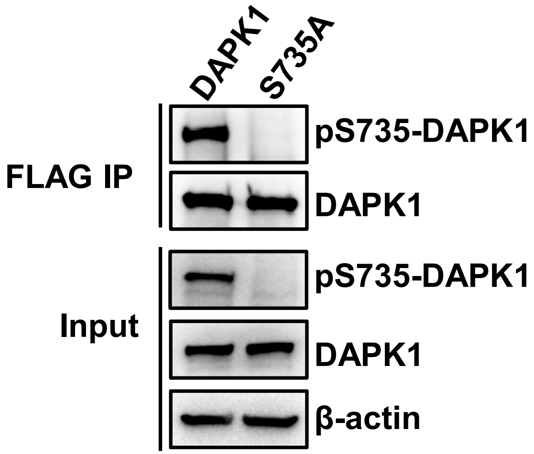

Figure S5 Gan et al.
